# Long-Term Outcomes of Endovascular Aneurysm Repair in Patients Aged ≤70 Years

**DOI:** 10.3400/avd.oa.23-00072

**Published:** 2024-02-06

**Authors:** Toshihiro Onohara, Nobuhiro Handa, Masakazu Kawasaki, Fuminori Kasashima, Tetsuya Saito, Teruya Nakamura, Dai Une, Mikizo Nakai, Suguru Shiraya, Kazuki Maeda, Katsuhiko Imai, Tsuyoshi Yamamoto, Yasushi Shimoe, Minoru Okamoto, Yoshikazu Kawazu, on behalf of National Hospital Organization Network Study Group in Japan for Abdominal Aortic Aneurysm

**Affiliations:** 1 Department of Vascular Surgery, Kyushu Medical Center, Fukuoka, Fukuoka, Japan; 2 Department of Cardiovascular Surgery, Nagara Medical Center, Gifu, Gifu, Japan; 3 Department of Cardiovascular Surgery, Hokkaido Medical Center, Sapporo, Hokkaido, Japan; 4 Department of Cardiovascular Surgery, Kanazawa Medical Center, Kanazawa, Ishikawa, Japan; 5 Department of Cardiovascular Surgery, Osaka National Hospital, Osaka, Osaka, Japan; 6 Department of Cardiovascular Surgery, Osaka Minami Medical Center, Kawachinagano, Osaka, Japan; 7 Department of Cardiovascular Surgery, Okayama Medical Center, Okayama, Okayama, Japan; 8 Department of Cardiovascular Surgery, Hamada Medical Center, Hamada, Shimane, Japan; 9 Department of Cardiovascular Surgery, Higashihiroshima Medical Center, Higashihiroshima, Hiroshima, Japan; 10 Department of Cardiovascular Surgery, Kure Medical Center, Kure, Hiroshima, Japan; 11 Department of Cardiovascular Surgery, Iwakuni Medical Center, Iwakuni, Yamaguchi, Japan; 12 Department of Cardiovascular Surgery, Shikoku Medical Center for Children and Adults, Zentsu¯ji, Kagawa, Japan; 13 Department of Cardiovascular Surgery, Kumamoto Medical Center, Kumamoto, Kumamoto, Japan; 14 Department of Cardiovascular Surgery, Kagoshima Medical Center, Kagoshima, Kagoshima, Japan

**Keywords:** abdominal aortic aneurysm, endovascular aneurysm repair, open surgical repair, young patients

## Abstract

**Objectives:** The efficacy of endovascular aneurysm repair (EVAR) against abdominal aortic aneurysm (AAA) in younger patients remains unknown. Hence, the current study aimed to investigate whether the aneurysm-related mortality rate of EVAR is acceptable among patients aged ≤70 years.

**Methods:** Among 644 patients, 148 underwent EVAR (EVAR group), and 496 received open surgical repair (OSR group). The cumulative incidence rates of aneurysm-related death, any intervention, and serious aneurysm-related events after AAA repair were evaluated using the cumulative incidence function in the presence of competing risks.

**Results:** The EVAR group had higher prevalences of several comorbidities, and overall survival for the EVAR group was significantly inferior to that of the OSR group. The cumulative incidence rates of aneurysm-related death, any intervention, and serious aneurysm-related events at 5 years were 1.5%, 11.7%, and 6.4% in the EVAR group and 1.3%, 5.3%, and 5.9% in the OSR group, respectively. EVAR was not a significant prognostic factor of aneurysm-related mortality and serious aneurysm-related events. However, it was an independent poor prognostic factor of any intervention.

**Conclusion:** EVAR was not a significant prognostic factor of aneurysm-related mortality and serious aneurysm-related events. Therefore, it demonstrated acceptable procedure-related long-term outcomes, at least in high-risk young patients.

## Introduction

Endovascular aneurysm repair (EVAR) has become a standard alternative to open surgical repair (OSR) for the management of abdominal aortic aneurysm (AAA). Several representative multicenter randomized trials in the early era of EVAR showed that the postoperative mortality rate of EVAR was lower than that of OSR. However, the early survival advantage of EVAR decreased over time.^1^^–^^3)^ This catch-up phenomenon was partly attributed to aneurysm-related mortality caused by factors such as secondary aneurysmal sac rupture after EVAR.^1)^ Furthermore, reintervention was provided more frequently after EVAR than after OSR.^1^^–^^3)^ A low perioperative mortality after OSR may counteract the early survival benefit of EVAR, and the efficacy of EVAR may not be satisfactory in patients with a longer life expectancy. Therefore, EVAR may not be effective in younger patients. However, in a meta-analysis of randomized trials about EVAR versus OSR in the abovementioned studies, EVAR was found to demonstrate favorable outcomes compared with OSR at any time period in patients aged <72 years. Nevertheless, the result was not significant.^4)^ In particular, in the Open versus Endovascular Repair (OVER) trial, patients aged <70 years in the EVAR group demonstrated a higher survival rate than those in the open-repair group.^5)^ Thus, whether EVAR is effective in terms of long-term survival in younger patients remains unknown. The current study aimed to investigate whether the cause-specific mortality, particularly aneurysm-related mortality, of EVAR, is acceptable in patients aged ≤70 years.

## Patients and Methods

This retrospective study analyzed the outcomes of EVAR in a large cohort at 14 centers under the National Hospital Organization in Japan.^6)^ The research was conducted in accordance with the Declaration of Helsinki guidelines. The study protocol was approved by the central human rights ethical committee (17E052, May 24, 2017, Kyushu Medical Center) and by the institutional review board of each participating center. All data, a total of 115 variables, including preoperative, intraoperative, and postoperative variables, were collected as part of routine diagnosis and treatment.

### Patients

From 2007 to 2013, 2517 patients who underwent AAA repair were retrospectively registered. Among them, 811 and 1706 underwent EVAR and OSR, respectively. Of the 2517 patients, 88 presented with shock at the beginning of surgery, and 86 underwent OSR. All patients with shock vital were excluded from the analysis. Of the remaining 2429 patients, 644 (26.5%) were aged ≤70 years. Finally, 644 patients were included in the analysis. Among them, 148 (23.0%) underwent EVAR (EVAR group) and 496 (77.0%) underwent OSR (OSR group).

Each patient underwent preoperative examination, including a multi-detector computed tomography scan, and the surgical procedure (EVAR or OSR) was selected based on the decision of surgeons or endovascular therapists at each participating center. EVAR outside the instructions for use (IFU) was performed if acceptable. In the analysis, outside the IFU (OIFU) was used according to neck features such as short neck, large diameter, angulated neck, circumferential thrombus, calcified neck, and reversed taper. In Japan, the uses of commercial devices for EVAR were approved by the Ministry of Labor and Welfare of Japan in January 2007, and all patients underwent OSR prior to the introduction of EVAR at each center. The patients visited the outpatient clinic every 6–12 months, and routine computed tomography scan was performed at 1, 6, and 12 months after EVAR within the first year and annually thereafter. If OSR was performed, a CT scan was not routinely scheduled. However, it was performed as needed.

### Endpoints

The primary endpoint was aneurysm-related mortality after AAA repair. The secondary endpoints were any intervention after AAA repair and serious aneurysm-related events. The tertiary endpoints were all-cause mortality and cause-specific mortalities associated with cardiovascular events, cancer, or infectious disease.

The causes of mortality were divided into aneurysm-related events, cardiovascular events, cancer, infection, and others. Aneurysm-related mortality was defined as operative mortality after primary AAA repair or late intervention or mortality caused by late-onset aneurysm-related complications such as AAA rupture and graft infection. Next, operative mortality was defined as death within 30 days after surgery or mortality during the same hospitalization for surgery. Cardiovascular mortality was classified as follows: death from cardiac, cerebrovascular, thoracic or thoraco-abdominal aortic (dissection or aneurysm), and other cardiovascular diseases, such as peripheral vascular or pulmonary embolism; sudden death; and operative death after cardiac or aortic surgery except for aneurysm-related events. Cancer-related mortality included death caused by primary malignancy after AAA repair or recurrence of previous or concomitant malignancy prior to AAA repair. Infection-related mortality included death due to pneumonia, acute respiratory failure, and sepsis attributed to other infections except for graft infection.

Serious aneurysm-related events were defined as aneurysm-related mortality or need for major interventions, which included different therapies, but not percutaneous endovascular surgery under local anesthesia such as balloon angioplasty, stent placement, and coil embolization. During the analysis of a particular death or an aneurysm-related event, if patients died due to other causes or without experiencing aneurysm-related events, the occurrence of a competing risk event was considered, and it was not censored.^7)^

### Statistical analysis

Statistical analysis was performed using STATA 15 (StataCorp LLC, College Station, TX, USA). The chi-squared test or the Fisher’s exact test was used to compare categorical variables between groups. The Wilcoxon rank-sum test was utilized to compare continuous and ordinal variables. Overall survival rates were assessed using the Kaplan–Meier method, and the Cox proportional hazard model was applied to determine multivariate associations. The cumulative incidence of each death divided by a particular cause of death after repair was evaluated using the cumulative incidence function method because death caused by other causes was considered as a competing risk event and may overestimate the incidence of deaths if a particular death was treated as censored data.^7)^ Moreover, the cumulative incidence of any intervention or major aneurysm-related events was assessed using the cumulative incidence function method. Next, the Fine–Gray subdistribution hazard model was used to determine multivariate associations according to the incidence rates of particular deaths, any interventions, and severe aneurysm-related events based on the following factors: type of surgical procedure (OSR or EVAR), sex, smoking status, body mass index, American Society of Anesthesiologist (ASA) physical status classification, comorbidities (such as hypertension, coronary artery disease, cerebrovascular disease with neurological deficits, diabetes mellitus, chronic obstructive pulmonary disease on inhaled drug, and concomitant cancer), preoperative laboratory data (serum creatinine, hemoglobin levels, and white blood cell count), and neck characteristics (followed the IFU or outside the IFU).

## Results

Of the 644 patients, 587 (91.2%) were men, and 57 (8.9%) were women. Moreover, they were aged 34–70 (mean: 64.5) years. Out of these, there were 503 (78.1%; 148 from the EVAR group and 355 from the OSR group) patients, in whom neck characteristics were documented to classify inside the IFU or OIFU. **Table 1** shows the characteristics of patients and perioperative data between the EVAR and OSR groups. The prevalence of previous cerebrovascular disease, chronic obstructive pulmonary disease, cancer, and laparotomy was higher in the EVAR group than in the OSR group. Consequently, patients in the EVAR group were likely to be at high risk based on the ASA physical status classification system. Conversely, patients with OIFU were more prevalent in the OSR group than in the EVAR group. Furthermore, 35 (9.3%) patients in the OSR group required suprarenal aortic clamping during AAA repair.

**Table:** Table 1 Characteristics of patients and perioperative data.

	EVAR group (n = 148)	OSR group (n = 496)	P
Age (years)	65.4 ± 4.1	64.2 ± 5.2	0.0197
Sex			
Male	136 (91.9%)	451 (90.9%)	0.8691
Female	12 (8.1%)	45 (9.1%)	
BMI (kg/m^2^)			
Mean	23.8 ± 3.4	23.9 ± 3.2	0.7308
<22.0	19 (13.0%)	63 (12.8%)	1.0000
Previous or current smoker	116 (78.4%)	357 (72.0%)	0.1378
Current smoker	51 (35.5%)	144 (29.0%)	0.2217
Hypertension	117 (79.1%)	384 (77.4%)	0.7359
CAD	51 (34.5%)	178 (35.9%)	0.7701
CVD without neurological deficit	14 (9.5%)	44 (8.9%)	0.0122
CVD with neurological deficit	16 (10.8%)	20 (4.0%)	
Diabetes mellitus	31 (21.0%)	77 (15.5%)	0.1327
COPD on inhaled drug	8 (5.4%)	5 (1.0%)	0.0029
Liver cirrhosis	2 (1.4%)	4 (0.8%)	0.6251
Previous cancer	13 (8.0%)	22 (3.6%)	0.0002
Concomitant cancer	8 (5.4%)	4 (0.8%)	
Previous laparotomy	29 (19.6%)	62 (12.5%)	0.0428
Hemoglobin level (g/dL)			
Mean	13.7 ± 1.6	13.3 ± 1.7	0.0081
<12.0	18 (12.7%)	95 (19.5%)	0.0812
White blood cell count (/mm^3^)			
Mean	6568 ± 2356	6765 ± 2697	0.4627
>8000	28 (19.6%)	98 (20.0%)	1.0000
Creatinine level (mg/dL)			
Mean	1.01 ± 0.83	1.07 ± 1.00	0.2307
>1.20	14 (9.7%)	68 (13.8%)	0.2572
eGFR (mL/min/1.73 m^2^)			
Mean	67.7 ± 19.2	65.6 ± 20.5	0.1500
<45.0	15 (23.0%)	64 (13.0%)	0.4741
Shape of aneurysm: Fusiform	114 (77.0%)	435 (87.7%)	0.0022
Saccular	34 (23.0%)	61 (12.3%)	
Diameter of aneurysm (mm)	49.7 ± 9.4	52.7 ± 15.2	0.0001
Followed the IFU	135 (91.2%)	217 (61.1%)	<0.0001
Outside the IFU	13 (8.8%)	138 (38.9%)	
ASA: class 3 or higher	42 (28.4%)	86 (17.3%)	0.0047
Operative time (min)	146 ± 66	242 ± 97	<0.0001
Volume of blood transfused (mL)	83 ± 274	709 ± 795	<0.0001
Suprarenal aortic clamping		35 (7.1%)	
Device used in EVAR			
Excluder	69		
Zenith	43		
Endurant	19		
Power Link	9		
Others/unknown	8		

Categorical variables were listed as N (%). Continuous variables were expressed as mean ± standard deviation.

BMI: body mass index; CAD: coronary artery disease; CVD: cerebrovascular disease; COPD: chronic obstructive pulmonary disease; IFU: instructions for use regarding neck features; ASA: American Society of Anesthesiologist physical status classification system; EVAR: endovascular aneurysm repair; OSR: open surgical repair

### Overall survival after AAA repair

**Figure 1:**
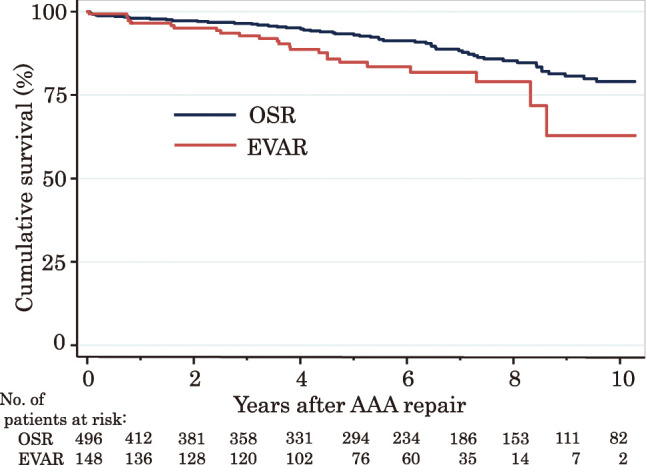
Fig. 1 Cumulative survival between the EVAR and OSR groups. The cumulative survival rates of the EVAR group were significantly lower than those of the OSR group. EVAR: endovascular aneurysm repair; OSR: open surgical repair; AAA: abdominal aortic aneurysm

Operative mortality occurred in one (0.7%) patient in the EVAR group and five (1.0%) in the OSR group. However, the results did not significantly differ between the two groups. During a mean follow-up period of 5.5 ± 3.5 years (median: 5.6 [interquartile range: 2.5–8.1] years), late mortality after AAA repair occurred in 75 patients (23 in the EVAR group and 52 in the OSR group). The causes of death in patients with late mortality after AAA repair in the EVAR and OSR groups were as follows: cardiovascular events (7/27), cancer (5/13), infectious disease (7/6), aneurysm-related event (1/1), and others or unknown cause (3/5). The cumulative survival rates of the EVAR and OSR groups were 96.6% ± 1.5% and 98.0% ± 0.7% at 1 year, 92.8% ± 2.2% and 96.5% ± 0.9% at 3 years, 84.8% ± 3.3% and 93.3% ± 1.3% at 5 years, and 79.0% ± 4.6% and 85.3% ± 2.1% at 8 years, respectively (**Fig. 1**). EVAR, OIFU, and concomitant cancer were significant poor prognostic factors of overall survival.

### Cause-specific mortality after AAA repair

**Figure 2:**
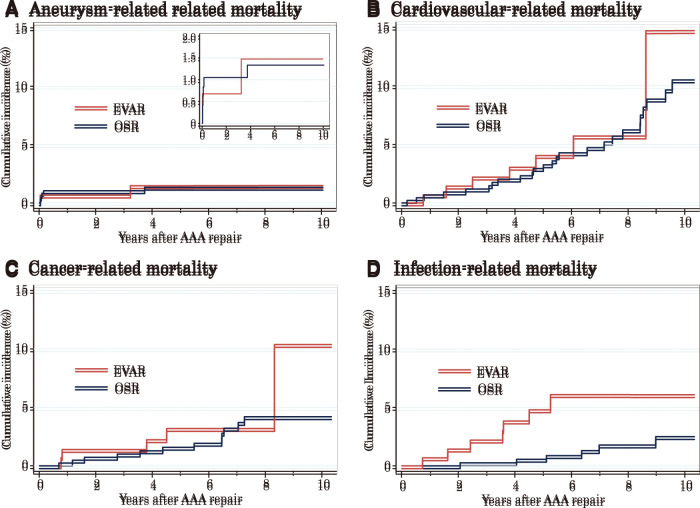
Fig. 2 Cumulative incidence of cause-specific mortality between the EVAR and OSR groups. Aneurysm-related mortality (**A**), cardiovascular-related mortality (**B**), cancer-related mortality (**C**), and infection-related mortality (**D**). The prevalence of infection-related mortality was significantly higher in the EVAR group than in the OSR group. No significant differences were found in terms of the cumulative incidence rates of aneurysm-, cardiovascular-, and cancer-related mortality between the two groups. EVAR: endovascular aneurysm repair; OSR: open surgical repair; AAA: abdominal aortic aneurysm

**Figure 2** presents the cumulative mortality rates due to aneurysm-related events, cardiovascular events, cancer, and infection between the EVAR and OSR groups. Regarding late aneurysm-related mortality, other than six operative deaths, one patient died 3.2 years after OSR for endoleak after EVAR, and one patient died due to aorto-duodenal fistula 3.7 years after OSR. No aneurysm-related mortality after 5 years was recorded in both groups. Consequently, the cumulative mortality rates of aneurysm-related events in the EVAR and OSR groups were 0.7% ± 0.7% and 1.1% ± 0.5% at 1 year, 0.7% ± 0.7% and 1.1% ± 0.5% at 3 years, and 1.5% ± 1.0% and 1.3% ± 0.6% at 5 years, respectively (**Fig. 2A**). Multivariate analysis showed that EVAR (**Table 2A**) and OIFU were not significant prognostic factors of aneurysm-related mortality.

**Table:** Table 2 Hazard ratios (95% confidence intervals) of aneurysm-related mortality, any intervention, and serious aneurysm-related events.

Parameters	Multivariate analysis		
	HR	95% CI	P
(A) Aneurysm-related mortality			
EVAR	1.10	0.08–15.79	0.944
IFU	3.15	0.55–17.97	0.196
CAD: none	3.54	0.83–15.11	0.088
CVD with neurological deficit	8.41	1.00–70.90	0.050
Diabetes mellitus	2.64	0.66–10.52	0.168
COPD on inhaled drug	17.71	1.83–170.87	0.013
Creatinine level >1.2 mg/dL	2.69	0.49–14.73	0.254
Hemoglobin level <12 g/dL	2.89	0.58–14.27	0.193
(B) Any interventions			
EVAR	2.59	1.27–5.27	0.009
Non-smoker	1.13	0.75–2.73	0.276
BMI ≥22.0 kg/m^2^	1.77	0.51–6.18	0.373
IFU	1.27	0.54–2.98	0.583
ASA: class 3 or higher	1.78	0.91–3.47	0.090
Hypertension	1.31	0.59–2.91	0.514
CAD	1.33	0.72–2.46	0.364
Creatinine level ≤1.2 mg/dL	2.75	0.64–1.18	0.173
(C) Serious aneurysm-related events			
EVAR	1.08	0.50–2.31	0.847
Female sex	2.67	1.06–6.76	0.038
BMI ≥22.0 kg/m^2^	2.57	0.71–9.26	0.150
CVD with neurological deficit	2.21	0.76–6.49	0.147
Diabetes mellitus	1.72	0.89–3.74	0.102
COPD	2.43	0.52–11.42	0.262
Creatinine level >1.2 mg/dL	1.56	0.64–3.81	0.324
Hemoglobin level ≥12 g/dL	2.83	0.85–9.40	0.089

HR: hazard ratio; CI: confidence interval; BMI: body mass index; IFU: instructions for use regarding neck features; CAD: coronary artery disease; CVD: cerebrovascular disease; COPD: chronic obstructive pulmonary disease; ASA: American Society of Anesthesiologist physical status classification system; EVAR: endovascular aneurysm repair

The cumulative incidence rates of cardiovascular mortality in the EVAR and OSR groups were 0.7% ± 0.7% and 0.7% ± 0.4% at 1 year, 2.2% ± 1.3% and 1.2% ± 0.5% at 3 years, 4.1% ± 1.8% and 2.9% ± 0.9% at 5 years, and 5.7% ± 2.4% and 6.2% ± 1.4% at 8 years, respectively (**Fig. 2B**). No significant prognostic factors were found for cardiovascular-related mortality. The cumulative incidence rates of cancer-related mortality in the EVAR and OSR groups were 1.4% ± 1.0% and 0.2% ± 0.2% at 1 year, 1.4% ± 1.0% and 1.0% ± 0.5% at 3 years, 3.2% ± 1.6% and 1.6% ± 0.6% at 5 years, and 3.2% ± 1.6% and 4.2% ± 1.2% at 8 years, respectively (**Fig. 2C**). Multivariate analysis revealed that EVAR was not a significant prognostic factor of cancer-related mortality. Moreover, cerebrovascular disease with neurological deficits and concomitant cancer were poor prognostic factors of cancer-related mortality. The cumulative incidence rates of infection-related mortality in the EVAR and OSR groups were 0.7% ± 0.7% and 0% at 1 year, 2.2% ± 1.3% and 0.3% ± 0.3% at 3 years, 4.8% ± 1.9% and 0.5% ± 0.4% at 5 years, and 6.1% ± 2.3% and 1.8% ± 0.8% at 8 years, respectively (**Fig. 2D**). EVAR, OIFU, and concomitant cancer were independent poor prognostic factors of infection-related mortality.

### Any intervention and serious aneurysm-related events after AAA repair

**Figure 3:**
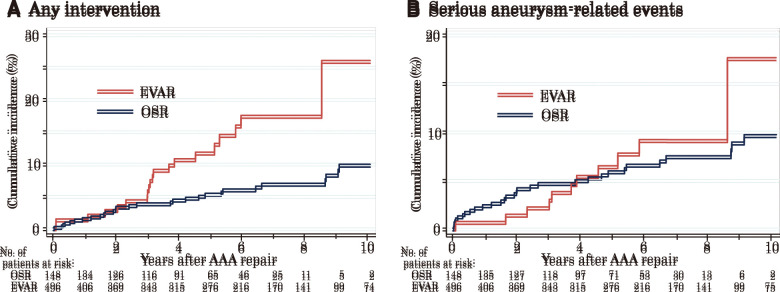
Fig. 3 Cumulative incidence of any intervention (**A**) and serious aneurysm-related events (**B**) between the EVAR and OSR groups. The cumulative incidence rates of any intervention were significantly higher in the EVAR group than in the OSR group. However, the cumulative incidence rates of serious aneurysm-related events did not significantly differ between the two groups. EVAR: endovascular aneurysm repair; OSR: open surgical repair; AAA: abdominal aortic aneurysm

During the follow-up period, 51 (7.9%) patients received interventions. These included the following: embolization for type 2 endoleak (n = 11), transperitoneal sacotomy or synthetic graft replacement (n = 6), repetitive EVAR including proximal or iliac stent-graft extension (n = 4), and femoro–femoral crossover bypass (n = 1) in the EVAR group; and repair of incisional hernia (n = 14), EVAR or iliac stent grafting (n = 4), percutaneous endovascular surgery for iliac artery stenosis (n = 3), repetitive OSR for pseudoaneurysm or graft infection (n = 2), lysis of intestinal adhesion for ileus (n = 2), femoro–femoral crossover bypass (n = 2), and thrombectomy (n = 2) in the OSR group. The cumulative incidence rates of any intervention in the EVAR and OSR groups were 1.4% ± 1.0% and 1.6% ± 0.6% at 1 year, 5.1% ± 1.9% and 3.9% ± 0.9% at 3 years, 11.7% ± 2.9% and 5.3% ± 1.1% at 5 years, and 17.4% ± 3.8% and 6.9% ± 1.4% at 8 years, respectively (**Fig. 3A**). EVAR was an independent poor prognostic factor of any intervention after AAA repair (**Table 2B**). The cumulative incidence rates of serious aneurysm-related event in the EVAR and OSR groups were 0.7% ± 0.7% and 2.4% ± 0.7% at 1 year, 2.2% ± 1.2% and 4.7% ± 1.0% at 3 years, 6.4% ± 2.2% and 5.9% ± 1.2% at 5 years, and 9.1% ± 2.9% and 7.5% ± 1.4% at 8 years, respectively (**Fig. 3B**). Multivariate analysis revealed that EVAR was not a significant prognostic factor of serious aneurysm-related events (**Table 2C**).

## Discussion

The major finding of the current study was that the cumulative aneurysm-related mortality was similar between the EVAR and OSR groups, while the EVAR group showed a significantly lower overall survival rate than the OSR group. In the EVAR group, this inferior survival was attributed to a higher rate of infection-related mortality and was probably secondary to higher prevalences of several comorbidities. EVAR might be selected for patients who appeared to be a high operative risk. Because the Kaplan–Meier method, in which deaths by other causes were treated as censored data, may overestimate the cumulative incidence rates of aneurysm-related mortality, the cumulative incidence function method, in which deaths by other causes were treated as competing risks, was used to calculate the cumulative incidence rates of aneurysm-related deaths. These findings suggested that EVAR is effective for high-risk young patients not only in terms of early but also for long-term outcomes.

In a meta-analysis of representative multicenter randomized trials in the early era of EVAR, such as the EVAR-1, DREAM, OVER, and ACE trials, the aneurysm-related mortality of the EVAR group was inferior to that of the OSR group within the first 30 days. However, after 3 years, a significant disadvantage for the EVAR group was found (hazard ratio: 5.16).^4)^ The EVAR-1 trial showed that the EVAR group demonstrated a significantly higher aneurysm-related mortality rate after 4 years than the OSR group (0.9 and 1.3 versus 0.2 and 0.2 deaths per 100 person-years in the EVAR and OSR groups within 4–8 years and after 8 years, respectively).^1)^ However, the overall aneurysm-related mortality was similar between the two groups (1.1 versus 0.9 deaths per 100 person-years in the EVAR and OSR groups, respectively).^1)^ By contrast, the current study showed that no early advantage exists in terms of operative mortality in the EVAR group and that the cumulative incidence rates of aneurysm-related mortality were similar between the EVAR and OSR groups (1.5% versus 1.3% at 5 years). Further, in the current study, the aneurysm-related mortality among the younger population might be relatively lower than in the EVAR-1 trial. Nolz et al. assessed late-onset stent graft-related endoleak, specifically type 1 and 3 endoleak, among 279 patients who underwent EVAR.^8)^ Results showed that 8.6% of patients presented with late-onset stent graft-related endoleak, and the significant risk factors were age, female sex, and left iliac sealing diameter. Candell et al. revealed that late aneurysm rupture after EVAR occurred after 30 postoperative days in 15 of 1736 patients. Moreover, the median time from EVAR to late rupture was 31.1 months, and the significant prognostic factors were age 80–89 years and symptomatic or rupture as the initial indication for EVAR.^9)^ In another recent report by their study group, the overall freedom rates from aneurysm-related mortality and from major adverse events or major reintervention were 96.4% and 84.0%, respectively. Further, female sex, age 80–89 years, urgent EVAR, and any major reintervention were the significant predictors of aneurysm-related mortality.^10)^ Therefore, age might be a major prognostic factor of aneurysm-related mortality after EVAR, and the prevalence of aneurysm-related mortality after EVAR may be relatively or negligibly low among younger patients.

Based on the abovementioned meta-analysis of multicenter randomized trials comparing the outcomes of EVAR or OSR, no significant differences were found in terms of the long-term overall survival rates between the EVAR and OSR groups.^4)^ Moreover, in another recent meta-analysis of the long-term outcomes of EVAR versus OSR, the long-term mortality did not significantly differ between the two groups. However, reintervention and secondary rupture were prevalent in the EVAR group.^11)^ The previous meta-analysis performed a subgroup analysis of overall mortality according to age, and EVAR was favorable in any time period among patients aged <72 years (hazard ratio: 0.83; 95% confidence interval [CI]: 0.66–1.04; P = 0.149). However, the results did not significantly differ.^4)^ The OVER trial showed that the survival rate after EVAR increased among patients aged <70 years (hazard ratio: 0.65; 95% CI: 0.43–0.98; P = 0.04).^5)^ In previous retrospective studies, Lee et al. assessed the long-term outcome of 169 patients aged ≤60 years (n = 119, OSR; n = 50, EVAR), and the results showed that the long-term survival was similar between the two groups.^12)^ The cumulative survival rates at 1, 5, and 10 years were 98%, 86%, and 54% in the EVAR group and 96%, 88%, and 75% in the OSR group, respectively. In addition, Reitz et al. showed that the long-term survival was similar among 553 patients aged <70 years between the groups (n = 204, EVAR; n = 49, OSR).^13)^ The cumulative survival rates at 2, 4, and 6 years were 97%, 95%, and 90% in the EVAR group and 100%, 97%, and 88% in the OSR group, respectively. However, in the current study, the overall survival rate in the EVAR group was significantly lower than that of the OSR group. The cumulative survival rates at 3, 5, and 8 years were 92.8%, 84.8%, and 79.0% in the EVAR group and 96.5%, 93.3%, and 85.3% in the OSR group, respectively. Our results were similar to those in the study by Lee et al. However, the long-term survival of the EVAR group was inferior to that in the study of Reitz et al. In terms of the characteristics of patients, a history of cardiovascular disease and concomitant cancer may be prevalent among patients in the EVAR group in our study, compared with the study by Reitz et al. Therefore, these factors affected the loss of overall survival in the current study. By contrast, the prevalence of coronary artery disease in our study population was lower. However, coronary artery disease was not a significant prognostic factor of overall survival in our study. EVAR has been performed in Japan since June 2007, when the use of commercial devices for EVAR was approved by the Ministry of Labor and Welfare of Japan. Our study cohort was collected in the early era of EVAR in Japan, and the prevalence of EVAR was less frequent among younger patients. Therefore, the selection of EVAR for younger patients could have been determined cautiously, and EVAR might have been performed on patients with frailty. A possible explanation was that EVAR was a significant prognostic factor of mortality due to infection but not cardiovascular- or malignancy-related mortality.

Among young patients, EVAR has several benefits compared to OSR. Early postoperative recovery after EVAR contributes to early social reintegration, and it is critical for the working generation. One of the postoperative complications after AAA repair is sexual dysfunction, which decreases the quality of life among younger patients. A review elucidating sexual dysfunction after AAA repair enumerated the causes of sexual dysfunction as follows: atherosclerosis and its risk factors, pelvic blood flow alternations via arterial clamping or hypogastric artery ligation, micro-embolization during surgery, hypogastric plexus injury, and medications such as anti-hypertensive drugs and statins.^14)^ Because EVAR can prevent hypogastric plexus injury and the use of a bifurcated iliac stent-graft can preserve the hypogastric artery, EVAR may be advantageous in preventing postoperative sexual dysfunction.

The current study exhibited several limitations. That is, it was retrospective in nature, and the selection of the primary and secondary procedures and the timing and indication of late intervention were dependent on each institution’s policy or decision of the physicians. Moreover, the study population might not be large enough to determine different prognostic factors conclusively because our study was performed in the early era of EVAR in Japan, and this procedure might have been performed less frequently among younger patients. Additionally, in the study, the follow-up period was not sufficiently long to detect late aneurysm-related deaths or events, as it was reported that aneurysm-related mortality occurred even after 8 years following EVAR.^1)^ Nevertheless, prospective randomized studies must be conducted to further assess whether EVAR is durable or valid among younger patients.

## Conclusions

We investigated the incidence and prognostic factors of cause-specific mortality after AAA repair among patients aged ≤70 years. Results showed that EVAR was not a significant prognostic factor of mortality caused by aneurysm-related events, although patients who underwent EVAR had a higher prevalence of several comorbidities and presented with an inferior overall survival compared to patients who underwent OSR. Thus, EVAR showed acceptable procedure-related long-term outcomes, at least in high-risk younger patients.

## Acknowledgments

The authors thank Junji Kishimoto, PhD, of Kyushu University Hospital, for statistical advice and Enago (www.enago.jp) for the English language review.

## Author Contributions

Study conception: TO

Data collection: all authors

Analysis: TO

Investigation: TO and NH

Manuscript preparation: TO and NH

Funding acquisition: NH

Critical review and revision: all authors

Final approval of the article: all authors

Accountability for all aspects of the work: all authors.

## Disclosure Statement

The authors have no potential conflicts of interest to disclose.
